# Research Note: Genome-wide association study for natural antibodies and resilience in a purebred layer chicken line

**DOI:** 10.1016/j.psj.2022.102312

**Published:** 2022-11-04

**Authors:** Harmen P. Doekes, Henk Bovenhuis, Tom V.L. Berghof, Katrijn Peeters, Jeroen Visscher, Han A. Mulder

**Affiliations:** ⁎ Wageningen University & Research Animal Breeding and Genomics, PO Box 338, 6700 AH Wageningen, The Netherlands; † Technical University of Munich, TUM School of Life Sciences, Reproductive Biotechnology, 85354 Freising, Germany; ‡ Hendrix Genetics B.V., P.O. Box 114, 5830 AC Boxmeer, The Netherlands

**Keywords:** GWAS, chicken, resilience, variance, natural antibody

## Abstract

Resilience is the capacity of an animal to be minimally affected by disturbances or rapidly return to the state pertained before exposure to a disturbance. Resilience indicators can be estimated from longitudinal production data, using deviations of observed from expected production levels. One component of resilience is disease resilience, which includes general disease resistance. Natural antibodies (**NAbs**) are an indicator trait for general disease resistance. The aim of this study was to perform a genome-wide association study (**GWAS**) for resilience indicators and NAbs in a Rhode Island purebred layer line and study potential overlap in genomic regions detected for these traits. For 2,494 hens, deviations (i.e., differences) between observed weekly egg production and expected weekly egg production were calculated. Resilience indicators were then defined as the natural logarithm of the variance of deviations, skewness of deviations, and lag-one autocorrelation of deviations. For a subset of 1,221 hens genotyped with the 60 K Illumina SNP BeadChip, NAbs binding keyhole-limpet hemocyanin were available (isotypes IgM and IgG). Heritabilities, estimated with a linear mixed animal model, were 0.39 for IgM and 0.20 for IgG, and ranged from 0.03 to 0.18 for the resilience indicators. No significant associations were found in the GWAS, except for a single chromosomal region for the skewness of egg deviations in wk 25 to 83 of the laying period. The absence of significant peaks for NAbs and resilience indicators suggests that there are no genes with major effect and that the traits are likely under polygenic control in this line.

## INTRODUCTION

Livestock are continuously exposed to environmental disturbances such as fluctuations in temperature or pathogens. Some animals can deal better with these disturbances, that is, are more resilient, than others. Resilience can be defined as an animal's capacity to be minimally affected by disturbances or rapidly return to the state pertained before exposure to a disturbance (Berghof et al., 2019).

Measuring general resilience of animals is challenging, but the increasing availability of longitudinal production data allows estimating resilience indicators based on deviations of observed from expected production levels. Such resilience indicators have been found to be heritable in various species, with heritability estimates ranging from 0.01 to 0.26 (Putz et al., 2019; Poppe et al., 2020; Bedere et al., 2022).

General resilience can be divided into different components such as heat stress resilience and disease resilience. Disease resilience can further be decomposed into disease resistance and disease tolerance. To evaluate an animal's general disease resistance, levels of natural antibodies (**NAbs**) can be used as indicator trait (Chen et al., 2020; Reyneveld et al., 2020). NAbs are immunoglobulins that are produced without previous exposure to a foreign antigen and have been shown to be heritable and associated with survival (Star et al., 2007; Berghof et al., 2018). Given the favorable relationship between NAbs and disease resilience, it is expected that favorable relationships exist between NAb-levels (as indicator for disease resistance) and general resilience.

Single nucleotide polymorphism (**SNP**) data can be used to identify genomic regions affecting NAb-levels and resilience (Berghof et al., 2018; Chen et al., 2020). In a purebred White Leghorn line, Berghof et al. (2018) found a region on chromosome 4 with a strong association with NAb IgM levels. Identification of such regions in other purebred layer lines would provide further insight in the genetic architecture underlying resilience, which may facilitate genetic improvement for resilience in these lines.

The aim of this study was to identify genomic regions influencing NAbs and resilience indicators in a Rhode Island layer line, and study potential overlap in genomic regions affecting these traits. We first determined NAbs binding keyhole-limpet hemocyanin (**KLH**) with an indirect 2-step ELISA and calculated resilience indicators from longitudinal egg production data. We then estimated genetic parameters and performed a genome-wide association study (**GWAS**).

## MATERIALS AND METHODS

### Animals

This study used data from a 2018 batch of a purebred Rhode Island line of Hendrix Genetics (Boxmeer, the Netherlands). Hens within this batch were hatched at 3 different time points, with 2-wk intervals. At 15 to 19 wk old, hens were housed individually. Hens were kept according to standard Hendrix Genetics protocols. The entire batch with phenotypes (n = 2,494 hens) was used to estimate genetic parameters for resilience indicators and NAbs. A subset of hens (n = 1,221) was genotyped and was used for the GWAS. Most of the genotyping was performed as part of the SusTradeoff project, in which random maternal families were selected and 5 random hens per maternal family were genotyped.

### Genotypes

Hens were genotyped with the 60 K Illumina SNP BeadChip. Genomic positions for the Gallus_gallus-5.0 assembly were available for 62,607 SNPs. SNPs were remapped to the GRCg7b assembly with the NCBI Genome Remapping Service (https://www.ncbi.nlm.nih.gov/genome/tools/remap), resulting in 62,435 successfully remapped SNPs. SNPs with more than 10% of genotypes missing were discarded (n = 1,030). When there were less than 10 hens for a genotype of a SNP, genotypes for that class were set to missing. For the Z-chromosome, heterozygous genotypes were also set to missing, since hens have only one Z-chromosome and thus a single allele for most SNPs. If a SNP had less than 10 hens for 2 of the 3 genotype classes, the entire SNP was discarded (n = 10,751). The remaining 50,654 SNPs were quite evenly distributed across the genome, although the micro-chromosomes 29 through to 33, micro-chromosomes 35 through to 39 and the W-chromosome were not covered.

### Phenotypes: NAbs and Resilience Indicators

Blood plasma samples were collected from the hens between 16 and 20 wk of age. Keyhole-limpet hemocyanin (KLH)-binding IgM and IgG NAbs were determined in plasma samples using an indirect 2-step ELISA. A detailed description of the procedure is provided by Berghof et al. (2018). The mean (SD) antibody titer for the 1,221 hens was 6.85 (1.21) for IgM and 6.03 (1.33) for IgG.

Resilience indicators were calculated based on the deviations (i.e., the differences) of a hen's observed weekly egg production from her expected weekly egg production, where the expected production was set to the batch mean (i.e., the mean of 2,494 hens). Egg production per hen was registered on a 1- to 4-day interval from the start of individual housing (at 15–19 wk of age) until the end of her life (i.e., at the end of laying period at 92 to 96 wk of age, unless she died or was euthanized earlier). The mean weekly egg production per hen was highest at 29 wk of age (6.8 eggs) and decreased until 92 wk of age (to 4.9 eggs). In very few cases, more than 7 eggs in a week for a hen were registered, which could be due to 1) a hen producing 2 eggs on a day, 2) a mismatch between the timing of egg laying and egg collection, for example, a hen laying a first egg after egg collection on d 1 and a second egg before collection on d 2, or 3) a registration error. Resilience indicators were the natural logarithm of the variance of deviations (**LNvar**), the skewness of deviations (**Skew**), and the lag-one autocorrelation of deviations (**Rauto**). A detailed description of these measures is provided by Berghof et al. (2019). Resilience indicators were calculated for 2 periods: from 25 wk of age to 83 wk of age (25–83) and from 83 wk of age to end of life (83-end). These periods were considered separately, because historically the breeding program focused on improving egg production up to 83 wk of age, while nowadays the focus is on prolonging the laying period (to > 100 wk of age). This may have resulted in differences in genetic variation underlying these traits. Records were set to missing when a hen laid less than 20 eggs in a period (n = 52 for period 25-83 and n = 373 for period 83-end). The mean (SD) of resilience indicators for the hens in the GWAS was 1.03 (1.65) for LNvar 25-83, 1.45 (1.96) for LNvar 83-end, −1.24 (1.33) for Skew 25-83, −0.45 (0.71) for Skew 83-end, 0.31 (0.30) for Rauto 25-83 and 0.04 (0.35) for Rauto 83-end.

### Estimation of Genetic Parameters

For each trait, genetic parameters were estimated with linear mixed models in ASReml 4.1 (Gilmour et al., 2015) using the entire batch of 2,494 laying hens. For NAbs, the following model was used:

$$y_{ijk}=\mu +plate_{i}+age_{j}+animal_{k}+e_{ijk}$$

where $plate_{i}$ is the fixed effect of the $i^{th}$ plate used for analyzing NAbs (146 levels), $age_{j}$ was the age at which NAb-levels were determined (4 levels, namely 112, 123, 124, or 138 d), $animal_{k}$ is the random additive genetic effect of the $k^{th}$ hen and $e_{ijk}$ is the random error term. We excluded hatch week from this model, because it was highly confounded with $age_{j}$ (where all hens from first hatch week had $age_{j}$ of 112 d, hens from second hatch week had $age_{j}$ of 123 or 124 d, and hens from the third hatch week had $age_{j}$ of 138 d). The animal-effects were assumed to be distributed as $N(0,A\sigma_{a}^{2}$), where $\sigma_{a}^{2}\;$is the additive genetic variance and **A** the pedigree-based relationship matrix. The error terms were assumed to be distributed as $N(0,I\sigma_{e}^{2}$), where $\sigma_{e}^{2}\;$is the residual variance and **I** an identity matrix.

For resilience indicators, the following model was used:

$$y_{ijk}=\mu +hatch\_week_{i}+row_{j}+animal_{k}+e_{ijk}$$

where $hatch\_week_{i}$ is the fixed effect of the $i^{th}$ week of hatching (3 levels), $row_{j}$ is the fixed effect of the $j^{th}$ row in the barn (12 levels) and the other model terms were the same as in the model for NAbs. Likelihood-ratio tests were used to test for additive genetic effects different from zero.

Initially, also maternal effects were fitted, but since models without the maternal effect were found to have a better model fit (i.e., a lower Akaike's Information Criterion) and for the sake of simplicity, maternal effects were excluded from the final models.

### Genome-Wide Association Study

A GWAS was performed for each trait, by running a linear mixed model for each SNP in ASReml 4.1 (Gilmour et al., 2015). The same models from the genetic parameter estimation were used, except that an additional SNP-effect was fitted and a **G**-matrix using VanRaden's method 1 (2008) was used to account for population structure. In each model, the SNP was fitted as a categorical variable with one level per genotype. To reduce computation time, the ratio of genetic over residual variance was fixed to the ratio which was estimated for the entire batch of 2,494 laying hens.

Solutions for SNP effects and conditional F-statistics were obtained from the ASReml output, and corresponding *P*-values were computed. Genomic inflation factors were computed as the ratio of the observed median χ^2^ statistic over the expected median of the corresponding χ^2^ distribution under the null hypothesis (with 1 degree of freedom). To correct for multiple testing, Q-values were calculated using the “*qvalue*” package in R (Dabney et al., 2010). Genome-wide false discovery rates (**FDR**) of 10% and 5% were used as suggestive and significant thresholds, respectively.

## RESULTS AND DISCUSSION

### Heritability Estimates

There was significant genetic variance for all traits except Skew 83-end, for which the *P*-value for the likelihood-ratio test was 0.051. Heritability estimates (SE) of NAbs were 0.39 (0.05) for IgM and 0.20 (0.04) for IgG. Heritability estimates of the resilience indicators were 0.08 (0.03) for LNvar 25-83, 0.14 (0.04) for LNvar 83-end, 0.11 (0.03) for Skew 25-83, 0.03 (0.02) for Skew 83-end, 0.18 (0.04) for Rauto 25-83 and 0.08 (0.03) for Rauto 83-end. These values are in the same order of magnitude as those from Bedere et al. (2022), who reported heritabilities of 0.12 (0.01) for LNvar 25-83, 0.02 (<0.01) for Skew 25-83 and 0.08 (0.01) for Rauto 25-83 for 34,397 hens of the same line. Berghof et al. (in preparation) also found heritability estimates of 0.02 to 0.08 for the different resilience indicators in the same line. We expect that the differences are mostly due to the focus on a single batch in the current study, with consequently substantial SEs. Some differences may also exist because, in contrast to the previous studies, we removed records when hens lay <20 eggs in a period in the current study (thereby removing high autocorrelations for animals that hardly laid any eggs in a period; n = 52 for the period 25-83 and n = 373 for the period 83-end). In general, our results suggest that NAb titers and resilience indicators are heritable and can be improved through breeding.

### Interpretation of Resilience Indicators

**Figure 1:**
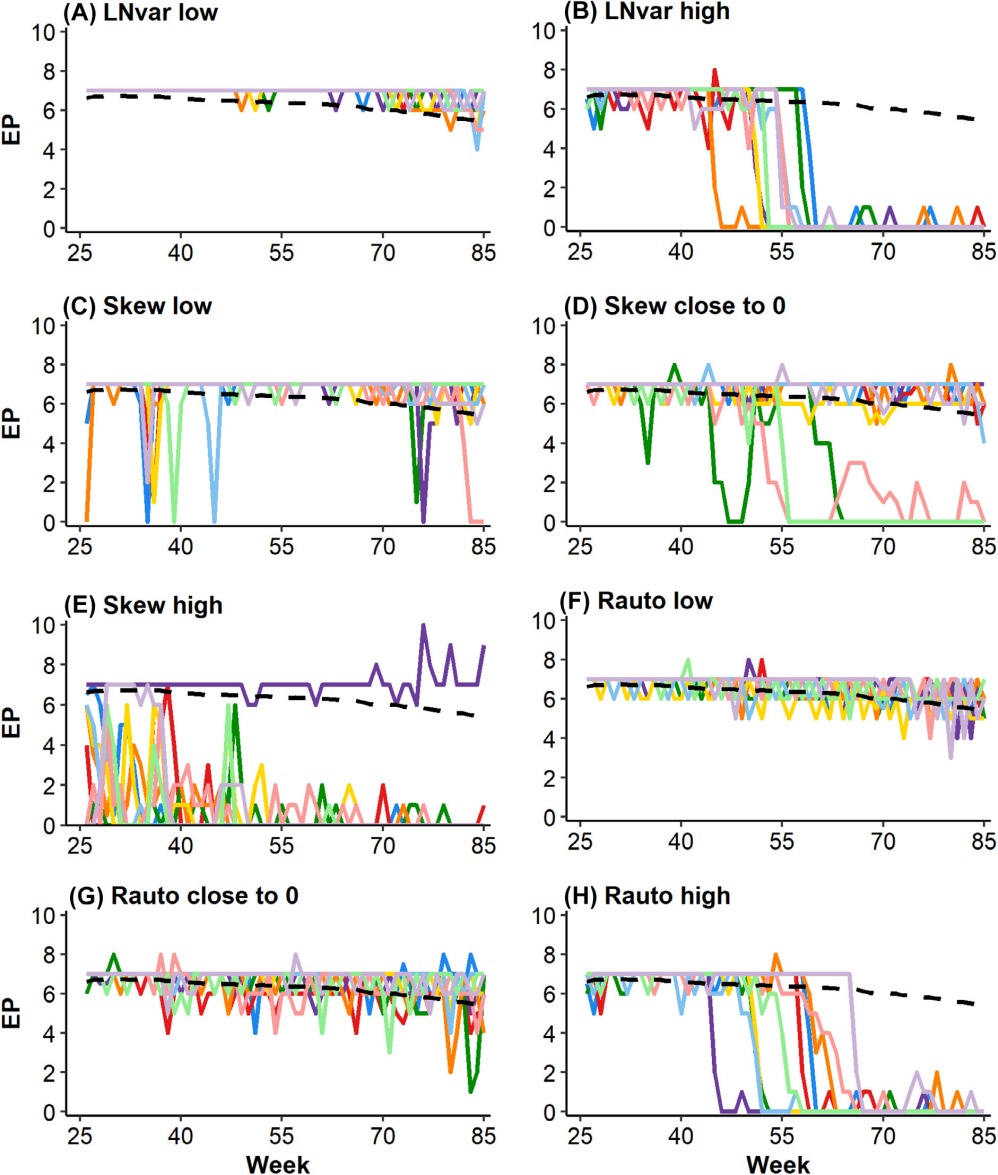
Egg production (EP) for wk 25–83 for animals with extreme values for the resilience indicators. The EP batch mean is the dashed black line. (A) Ten animals with the lowest LNvariance, (B) 10 animals with highest LNvariance, (C) 10 animals with lowest skewness, (D) 10 animals with skewness closest to zero, (E) 10 animals with highest skewness, (F) 10 animals with lowest autocorrelation, (G) 10 animals with autocorrelation closest to zero, and (H) 10 animals with highest autocorrelation.

Figure 1 shows the production curves for the 10 laying hens with the most “extreme” (i.e., highest, lowest or closest to zero) resilience values for the period 25 to 83. As illustrated in Figure 1 and discussed by Berghof et al. (2019) and Poppe et al. (2020), LNvar, Skew, and Rauto capture different aspects of resilience. Interpretation of the different indicators, however, is not straightforward. For example, a skewness of approximately 0 is expected for animals without disturbances or animals that are not influenced by disturbances, but may also occur for animals that show substantial fluctuations and have a production curve that differs a lot from the expected curve (Figure 1D). As long as the deviations form an approximate normal distribution around the animal's mean deviation, the skewness will be close to zero. A high LNvar is expected for animals that have large fluctuations in weekly production, but the highest LNvar values were found for hens with low laying persistency (Figure 1B). This is a consequence of using the batch mean for expected values. Similarly, an autocorrelation towards +1 (i.e., where subsequent deviations are more alike) may occur for animals affected by disturbances and with a slow recovery, but the highest Rauto values were found for animals with low persistency (Figure 1H). Even if an animal would constantly produce 7 eggs, it would have a Rauto of >0.95, simply because the batch mean decreases and therefore deviations increase over time and subsequent deviations become highly correlated. These limitations could be partly overcome by considering individual curves for the expected production, rather than using the batch mean. However, use of individual production curves prompts other questions regarding calculation and interpretation, such as how the height and shape of the expected curve (i.e., the potential maximum curve in absence of disturbances) for each animal should be determined, given that the observed production curves may be influenced by disturbances (Mertens et al., 2009; Poppe et al., 2020). Berghof et al. (in preparation) estimated resilience indicators based on individual expected laying curves (based on 0.7 quantile regression) and found for LNvar and Rauto moderate to very high genetic correlations with the indicators based on the batch mean, but not for Skew. Thus, the genetic ranking of animals for LNvar and for Rauto is similar when using the batch mean or when using individual expectations as “reference curve”. This is because LNvar and Rauto largely depend on fluctuations in deviations (and not on the absolute level of the reference curve). In general, the present and other studies demonstrate that there is not (yet) a golden standard for quantification of resilience. As recently discussed by Friggens et al. (2022), the evaluation of candidate indicators is now key to move toward operational measures of resilience.

### Genome-Wide Association Study

**Figure 2:**
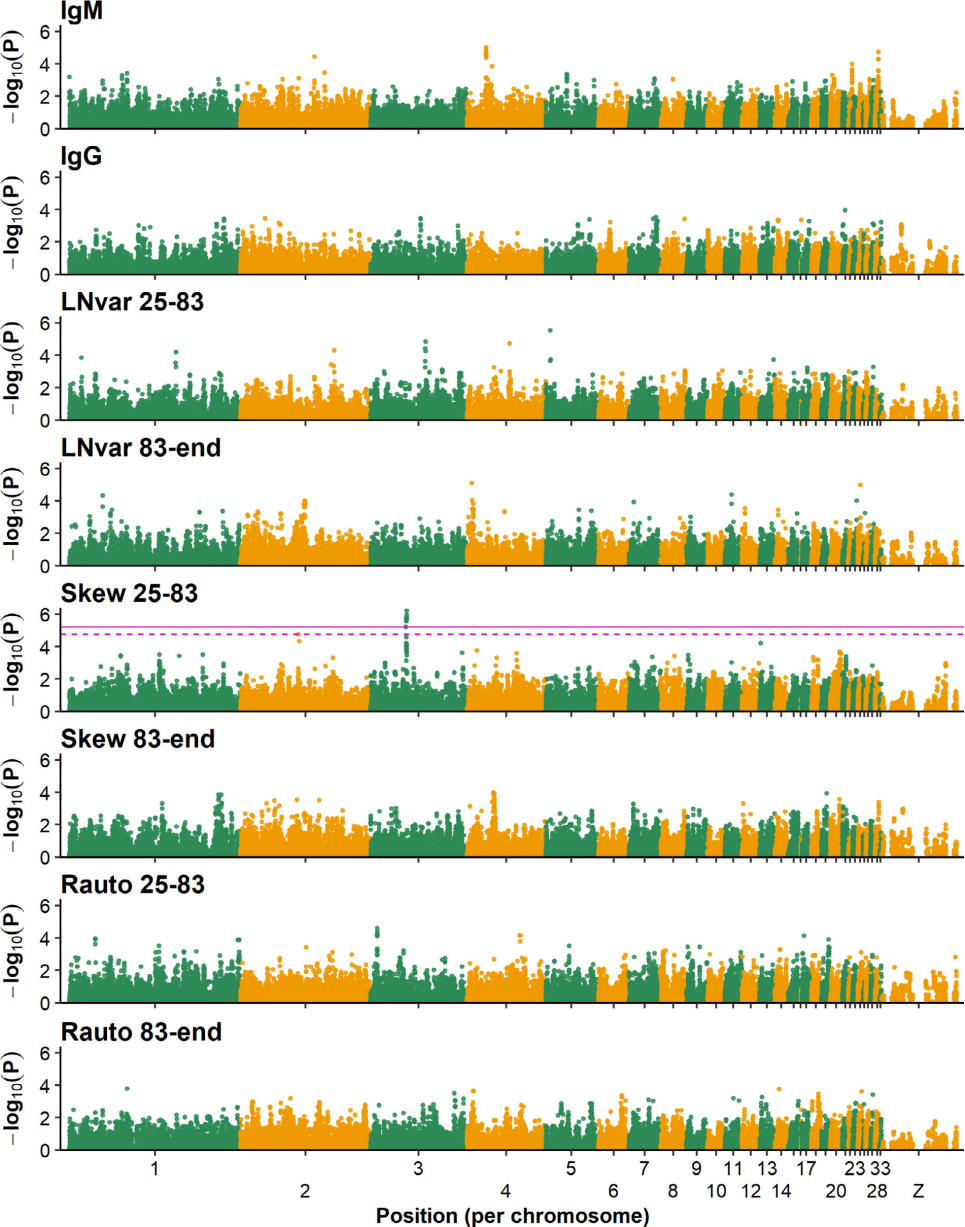
Significance of SNP effects for natural antibodies (IgM and IgG) and resilience indicators (LNvar, Skew and Rauto for two parts of laying period) from a single SNP GWAS. The horizontal purple lines are thresholds where SNPs on or above this line had a Q-value below 0.10 (dashed line) and below 0.05 (solid line). Absence of these lines implies that none of the SNPs was below the thresholds.

Genomic inflation factors were ≤1.05 for all traits, suggesting no inflation of *P*-values for the estimated SNP effects. There was a single peak with significant SNPs for Skew 25-83, between 40.7 and 41.4 Mb on chromosome 3 (Figure 2). Based on our knowledge, and given the complicated interpretation of skewness, no conclusive biological explanation for this association can be given. This region covered the genes *ERMARD, TCTE3, PHF10, C6orf120, WDR27, THBS2*, and *SMOC2* (https://www.ncbi.nlm.nih.gov/genome/gdv/). For none of these genes we can suggest a direct relationship with Skew 25-83, potentially due to a lack of annotation, although *SMOC2* is involved in wound healing and, thus, in recovery after cell stress. For all other resilience indicators, no significant or suggestive associations were found. This is in line with most GWAS on traits related to uniformity and resilience, which typically find few significant associations with small effects (Iung et al., 2020). To the best of our knowledge, our study is the first GWAS for resilience indicators based on longitudinal production data in laying hens.

For NAb titers, there were no significant or suggestive associations. For IgM, however, there were some peaks that did not pass the significance thresholds, for example, around 21.7-22.2 Mb on chromosome 4. The latter was not the same region as that which was previously identified for the “WA” White Leghorn line (Berghof et al., 2018). The region identified by Berghof et al. (2018) was located between 68.6 and 71.5 Mb (between 69.5 and 72.5 Mb on Gallus_gallus-5.0 assembly) and the lead SNP explained approximately 60% of the genetic variation of KLH-binding IgM. Through imputation to whole genome sequence, *Toll-like receptor family member 1A* (***TLR1A***) was identified as the major candidate gene, with the most likely causal variant at 69,038,449 bp (69,965,939 bp on Gallus_gallus-5.0). The *TLR1A* polymorphism appears not to be segregating in this Rhode Island (BD) line in the current study. In two other White Leghorn lines, the “WD” and W1” lines, the polymorphism was furthermore found not to be segregating based on sequence data (unpublished data). Thus, the *TLR1A* polymorphism appears to be specific for the WA line. Biscarini et al. (2010) performed a GWAS in a combined sample of 583 White Leghorn and Rhode Island hens with ∼1k SNP data, and reported various candidate SNPs on chromosomes 3, 4, 5, 7, 13 19, and Z for KLH-binding NAbs measured at 20, 40, or 65 wk of age. Their study was focused on across-line associations, considered total NAb titers (all isotypes) and the reported associations were also not very strong (the largest -log_10_(*P*-value) was 3.4).

There were no indications for genomic regions influencing both NAb titers and resilience indicators (Figure 2). Berghof et al. (in preparation) found nonsignificant genetic correlations, ranging from −0.27 to 0.56, between NAb titers and resilience indicators in a larger number of hens from the Rhode Island line. They also observed a moderate genetic correlation of 0.24 (SE = 0.12) between IgM and IgG levels. Thus, although a (genetic) relationship between NAbs and resilience might be expected, current evidence for this relationship is limited.

It should be noted that the limited coverage of some genomic regions, especially of micro-chromosomes 29 through to 33 and 35 through to 39, may have led to missing potential QTL in these regions. There are 568 known coding genes on the micro-chromosomes 29 through to 33 and 35 through to 39, which is ∼3% of the total number of known coding genes in the chicken genome (Ensemble, 2022). Follow-up studies with higher density information would be required to also capture potential signals in these regions of the genome.

Overall, the findings of this study suggest that both NAb titers and resilience indicators are heritable and have potential for genetic improvement of (disease) resilience. From the resilience indicators in this study, LNvar may be the most promising, given its considerable genetic variance and seemingly most intuitive interpretation. However, interpretation of none of the resilience indicators is straightforward and different resilience indicators capture different facets of resilience. Therefore, multiple resilience indicators might be needed to capture total resilience for management and breeding purposes. Last, both NAb titers and resilience indicators appear to have a polygenic basis, with no major genes identified in the Rhode Island line.
